# A clinical prediction nomogram for hemorrhoid risk in health screening populations in Beijing, China: a retrospective cohort study of 35,256 adults

**DOI:** 10.3389/fmed.2026.1846099

**Published:** 2026-09-28

**Authors:** Jia Wu, Keyang Zheng, Haixiang Liu, Li Yan

**Affiliations:** 1 Department of General Surgery, Beijing Nuclear Industry Hospital, Beijing, China; 2 Department of General Practice, Beijing Nuclear Industry Hospital, Beijing, China; 3 Health Examination Center, Beijing Nuclear Industry Hospital, Beijing, China; 4 Department of General Surgery, Beijing Tongren Hospital, Beijing, China

**Keywords:** biomarkers, cohort study, health screening, hemorrhoids, risk prediction

## Abstract

**Background:**

Hemorrhoids affect approximately 25% of the global population, yet validated risk prediction models for primary prevention are lacking. Routine laboratory biomarkers offer practical advantages for risk prediction in health screening settings. This study aims to identify independent risk factors for hemorrhoid incidence and develop a clinical prediction nomogram incorporating sex, age, and routine laboratory biomarkers in a large health-screening cohort.

**Methods:**

This retrospective longitudinal cohort study included 35,256 adults who underwent comprehensive health examinations at Beijing Nuclear Industry Hospital, China, between December 29, 2014, and December 16, 2025. Participants without prior hemorrhoid disease were followed for 60 months through subsequent screening visits. Cox proportional hazards regression identified independent predictors from 16 candidate variables including demographics and laboratory parameters. A nomogram was constructed and validated using discrimination analysis (C-index and time-dependent ROC), calibration curves, and decision curve analysis.

**Results:**

Over the 60-month follow-up period, 3,973 of the 35,256 participants (11.27%) developed incident hemorrhoids. Multivariable analysis identified five independent predictors: female sex (HR = 1.41, 95% CI: 1.28–1.55), older age (HR = 1.02, 95% CI: 1.02–1.03 per year), lower hemoglobin (HR = 0.99, 95% CI: 0.99–1.00 per g/L), higher triglycerides (HR = 1.05, 95% CI: 1.02–1.08 per mmol/L), and higher uric acid (HR = 1.001, 95% CI: 1.001–1.002 per μmol/L). The nomogram demonstrated acceptable discrimination (C-index: 0.772, 95% CI: 0.764–0.780), though it systematically underestimated observed risk across the predicted-probability range (∼0%–37%); decision curve analysis showed positive net benefit for threshold probabilities between approximately 5%–40%.

**Conclusions:**

This study identified five independent predictors of hemorrhoid incidence—sex, age, hemoglobin, triglycerides, and uric acid—and used them to develop one of the first nomograms integrating routine laboratory biomarkers for hemorrhoid risk prediction in health-screening populations. Using exclusively accessible, low-cost parameters, the model enables individualized risk stratification at routine health screening, facilitating targeted prevention strategies for high-risk individuals.

## 1 Introduction

Hemorrhoidal disease affects approximately one-quarter of the global population. A recent meta-analysis of 150 studies comprising 8,960,338 individuals reported a pooled point prevalence of 25.92%, a one-year prevalence of 21.65%, and a lifetime prevalence of 27.19% (1). Prevalence is higher in women (27.33%) than in men, and is highest in the African region (28.07%) (1). In unadjusted analyses, the disease has been associated with older age, obesity, pregnancy, diabetes, family history, constipation, and hypertension (1).

Population-based surveys in China report hemorrhoid prevalence of approximately 50% (2)—nearly double the global average—reflecting the combined effects of dietary patterns, sedentary occupations, and a rapidly rising metabolic disease burden. In China, where annual employer-sponsored health examinations cover more than 400 million adults (3) and routine biochemical and haematological panels are universally collected, no validated risk-prediction tool for incident hemorrhoidal disease currently exists. Despite this substantial disease burden and its impact on quality of life, validated risk-stratification tools for primary prevention remain absent. Developing such a tool in a large Chinese health-screening cohort is therefore both feasible and clinically pressing.

Genome-wide association study meta-analysis of 218,920 patients with hemorrhoidal disease and 725,213 controls demonstrated modest heritability and genetic correlation with gastrointestinal, neuroaffective, and cardiovascular domains. The same study identified 102 independent risk loci harbouring genes whose expression is enriched in blood vessels and gastrointestinal tissues (4). However, most evidence for traditional risk factors derives from cross-sectional designs susceptible to recall bias and reverse causation (5).

Routine laboratory biomarkers—in particular hemoglobin, triglycerides, and uric acid—are therefore the principal candidate predictors of interest in the present study, for three reasons: (i) they reflect the vascular and metabolic pathways implicated in hemorrhoidal pathogenesis (see conceptual framework, Figure 1); (ii) they are objectively measured and free from recall bias; by contrast, self-reported lifestyle factors such as dietary fiber intake, bowel habits, and physical activity have shown only modest, and in some analyses non-significant, associations with hemorrhoid risk in large screening-based cohorts (odds ratios approximately 0.78–0.83) (6), and are additionally subject to substantial recall bias and measurement error (7); and (iii) they are universally available in standard health-screening panels at no additional cost.

**Figure 1:**
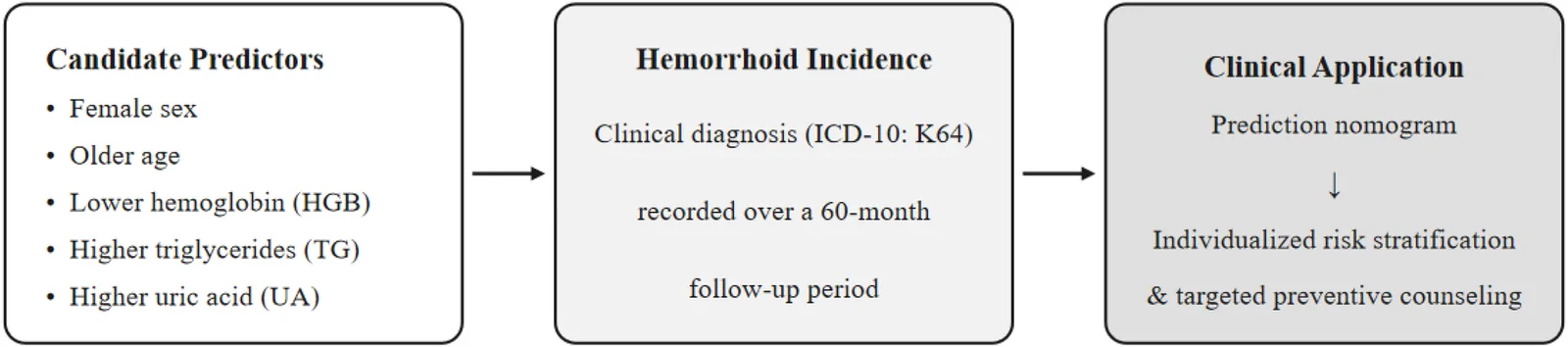
Conceptual framework of the hemorrhoid risk prediction model, showing the candidate predictors, the outcome (hemorrhoid incidence, ICD-10: K64), and the intended clinical application.

Hemorrhoidal disease involves abnormal, tortuous dilatation of the anorectal arteriovenous plexus (8). Dilated hemorrhoidal veins undergo segmentation, a vascular remodeling process linked to hemodynamic factors (9). Increased mast-cell density in hemorrhoid venous blood vessels suggests involvement in pathophysiology through granule release affecting vessel calibre, permeability, and thrombosis (4). The anorectal vascular plexus morphology and functional mechanisms directly impact the course of hemorrhoidal disease (10).

Hemoglobin levels may reflect chronic occult bleeding leading to anaemia, with resultant mucosal hypoxia impairing tissue-repair capacity. However, this directionality is not without ambiguity: lower baseline hemoglobin can also reflect occult bleeding from already-existing but undiagnosed hemorrhoidal vessels, and we return to this reverse-causation concern explicitly in the Discussion. Elevated triglycerides contribute to endothelial dysfunction and metabolic inflammation, compromising the vascular integrity of the hemorrhoidal plexus. Hyperuricaemia induces endothelial dysfunction through several pathways: it impairs insulin-stimulated endothelial nitric-oxide synthesis by interfering with insulin-receptor-substrate and PI3K/Akt signalling, decreases endothelial nitric-oxide synthase expression and activity, and directly inactivates nitric oxide (11). Hyperuricaemia thus serves as a key contributor to endothelial dysfunction in cardiovascular diseases (12). Impaired NO-dependent vasodilation compromises hemorrhoidal vascular function. Given these mechanistic links, longitudinal investigation of these biomarkers' predictive value in a large health-screening cohort is warranted.

This study aimed to identify independent risk factors for hemorrhoid incidence in a longitudinal health-screening cohort of 35,256 adults followed for 60 months, and to develop a clinical prediction nomogram incorporating sex, age, hemoglobin, triglycerides, and uric acid. To our knowledge, this is the first nomogram of its kind developed in a Chinese health-screening setting, and we contextualize its design and performance against indirect comparators such as the comprehensive colorectal-cancer risk model (13) and existing metabolic-syndrome and cardiovascular risk nomograms in the Discussion.

## 2 Methods

### 2.1 Data source

This study used de-identified secondary data routinely collected by the Health Examination Centre of Beijing Nuclear Industry Hospital and stored in the hospital's electronic medical-record system. No new data were collected for the purpose of this study. The data extraction window covered all comprehensive health examinations performed between December 29, 2014 and December 16, 2025.

### 2.2 Study design and population

A retrospective cohort study with longitudinal follow-up was conducted at Beijing Nuclear Industry Hospital, Beijing, China. The study employed a 60-month follow-up design to capture hemorrhoid incidence while balancing sufficient event accumulation against participant retention.

The inclusion criteria were as follows: (1) age between 20 and 70 years at baseline health examination, selected to exclude pediatric populations and very elderly individuals with multiple comorbidities that could confound hemorrhoid risk assessment; (2) availability of complete demographic and laboratory data at baseline; (3) no prior diagnosis of hemorrhoid disease at baseline; and (4) follow-up duration of at least 6 months to ensure adequate opportunity for outcome ascertainment. The exclusion criteria included: (1) participants with pre-existing hemorrhoid disease at baseline; (2) participants with a history of malignant tumors or prior malignant tumor surgery, as cancer and its treatments may independently affect hemorrhoid risk through mechanisms including pelvic surgery, radiation therapy, and altered bowel habits; (3) participants with incomplete baseline records for key variables; and (4) participants lost to follow-up before the first subsequent examination (follow-up duration <6 months). The cohort was drawn from an employer-sponsored occupational health-screening program in which pregnant employees are not scheduled for the mandatory annual examination; consequently, the cohort did not include pregnant women, and current pregnancy could not be evaluated as a covariate. Parity (history of childbirth), which was recorded for female participants, was therefore examined separately as the closest available proxy for reproductive history within this cohort.

The participant selection process proceeded sequentially (Figure 2): from an initial pool of 42,436 individuals aged 20–70 years with multiple follow-up records, 39,734 remained after excluding those with pre-existing hemorrhoid disease at baseline (*n* = 2,702 excluded); 39,320 remained after excluding those with a history of malignant tumors or prior malignant tumor surgery (*n* = 414 excluded); 38,814 remained after excluding those with follow-up duration less than 6 months (*n* = 506 excluded); and 35,256 participants were finally included after excluding those with incomplete baseline records for key variables (*n* = 3,558 excluded).

**Figure 2:**
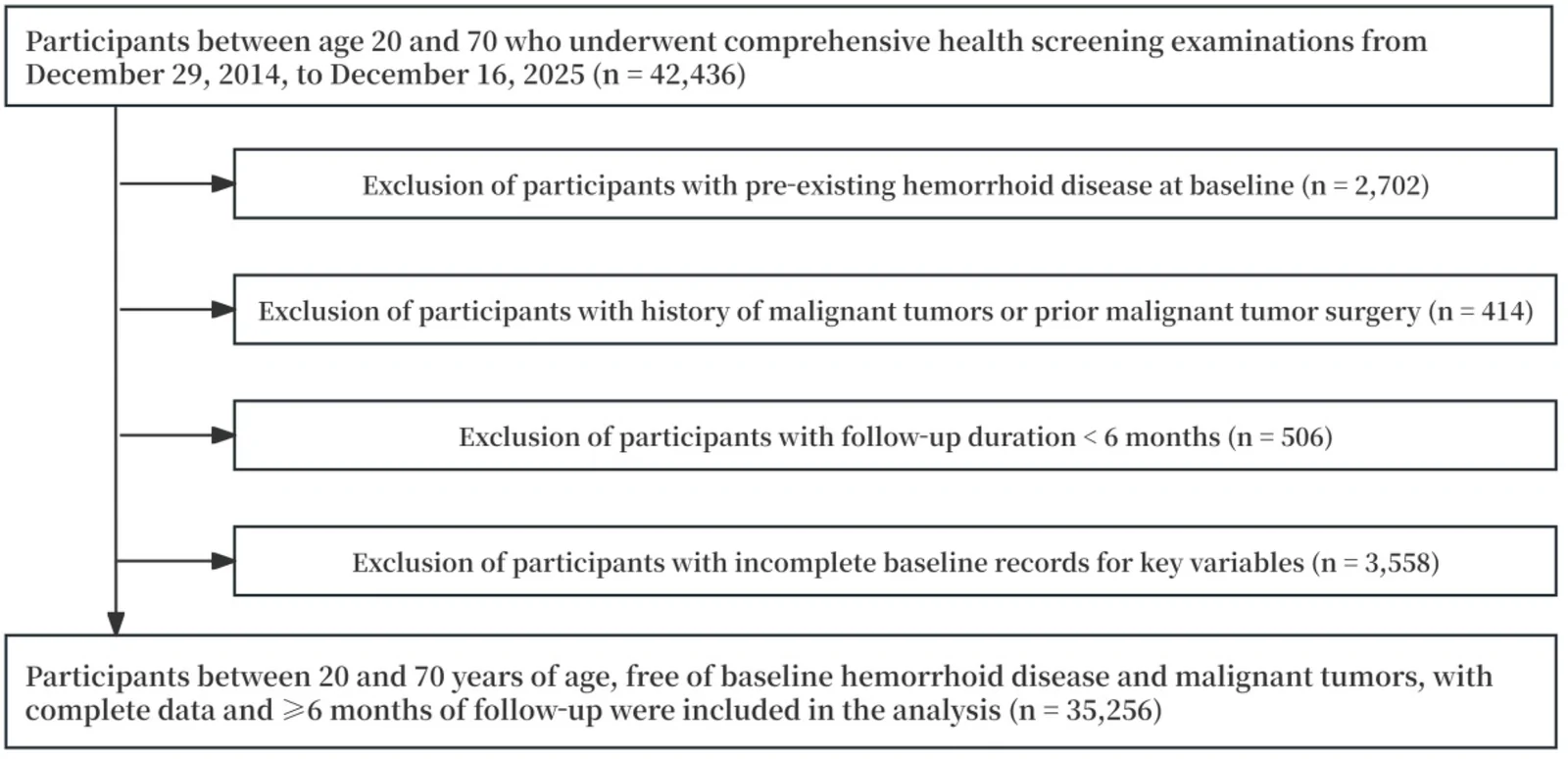
Flow diagram of participant selection.

Sample-size adequacy was confirmed *post hoc* using the events-per-variable (EPV) rule. With 3,973 events and 5 final predictors retained in the multivariable Cox model, the EPV was 794.6—far above the conventional EPV ≥10 threshold for Cox regression and the more conservative EPV ≥20 threshold recommended for clinical-prediction-model development.

This study was conducted in accordance with the Declaration of Helsinki and was approved by the Ethics Committee of Beijing Nuclear Industry Hospital. The requirement for informed consent was waived due to the retrospective nature of the study using de-identified health screening data.

### 2.3 Data collection and quality control

Data extraction from the hospital's electronic medical-record system was performed by two trained research assistants using a standardized SQL extraction script. Because all source fields are pre-coded—laboratory values, ICD-10 diagnoses, and demographic variables—extraction was largely deterministic. To verify reliability, a 10% random sample of records was independently re-extracted; agreement was 100% for laboratory values and 99.8% for diagnostic coding, with all discrepancies adjudicated by the senior author.

### 2.4 Variable definitions

Baseline data were collected during the initial health-screening visit and included demographic characteristics and laboratory measurements. Demographic variables comprised sex (male/female), age (years), and smoking status (current smoker/non-smoker). Smoking status was self-reported and did not distinguish former smokers or capture smoking intensity (e.g., pack-years). Laboratory variables included hemoglobin (HGB, g/L), white blood cell count (WBC, ×10^9^/L), platelet count (PLT, ×10^9^/L), body-mass index (BMI, kg/m^2^), low-density lipoprotein cholesterol (LDL-C, mmol/L), triglycerides (TG, mmol/L), alanine aminotransferase (ALT, U/L), aspartate aminotransferase (AST, U/L), creatinine (Cr, µmol/L), uric acid (UA, µmol/L), fasting plasma glucose (FPG, mmol/L), diastolic blood pressure (DBP, mmHg), and systolic blood pressure (SBP, mmHg). A total of 16 candidate predictor variables were considered for model development. Parity (any prior childbirth, coded as a binary variable) was additionally recorded for female participants. Because parity is structurally zero among male participants and therefore collinear with sex, it was not included among the 16 candidate predictors screened for the overall multivariable model; instead, it was examined in a sensitivity analysis restricted to female participants.

All laboratory measurements were obtained from fasting venous blood samples collected during the health examination, processed and analyzed using standardized procedures and internal quality-control protocols in the hospital's clinical laboratory, in accordance with established laboratory guidelines (14). BMI was calculated as weight (kg) divided by height squared (m^2^). Blood pressure was measured using a validated automated sphygmomanometer after participants had rested for at least 5 min in a seated position, in accordance with current hypertension-measurement guidelines (15).

### 2.5 Outcome definition

The primary outcome of this study was the occurrence of new-onset hemorrhoid disease during the follow-up period. At each health-screening visit, participants underwent a standardized physical examination performed by a surgeon, which included visual inspection and, where clinically indicated, digital examination of the perianal region; findings were recorded in a structured departmental examination summary within the electronic medical record, and consistency between these examination findings and the coded hemorrhoid outcome variable was confirmed during data verification. Hemorrhoid disease was identified based on clinical diagnosis documented during subsequent health-screening examinations or through linkage with outpatient and inpatient medical records, using the International Classification of Diseases, Tenth Revision (ICD-10) code K64 (hemorrhoids and perianal venous thrombosis), as defined in the WHO ICD-10 classification manual (16). The follow-up time for each participant was calculated from the date of the baseline health examination to the date of first hemorrhoid diagnosis (for cases) or the date of the last follow-up visit (for censored participants). During the study period, 3,973 participants (11.27%) developed new-onset hemorrhoid disease.

### 2.6 Statistical analysis

Following participant selection, the dataset underwent systematic quality control and imputation procedures. Missing data were observed across several demographic and laboratory variables. A pre-specified rule would have excluded any variable with more than 50% missingness from analysis; all retained variables fell below this threshold. For variables with missing values, multiple imputation was performed using Predictive Mean Matching (PMM), an approach that preserves the original data distribution while generating plausible values for missing observations. Numeric variables underwent format standardization, and categorical variables were label-encoded prior to imputation. The imputation process was iterated 5 times to ensure convergence and stability of estimates. After imputation, categorical variables were reverted to their original encoding, and the complete dataset was verified to contain no remaining missing values.

Baseline characteristics were compared between participants who developed hemorrhoid disease and those who did not. Continuous variables were described as mean ± standard deviation (SD) and compared using the independent-samples *t*-test or the Mann–Whitney *U* test, as appropriate. Categorical variables were expressed as frequencies and percentages and compared using the chi-square test or Fisher's exact test. Because statistical significance is strongly influenced by the large sample size, the standardized mean difference (SMD) was additionally calculated for each baseline variable to provide a sample-size-independent measure of the magnitude of between-group imbalance.

Univariate Cox proportional hazards regression was first performed to evaluate the association between each candidate variable and the risk of new-onset hemorrhoid disease. Variables with a *P* value <0.05 in the univariate analysis were subsequently entered into the multivariate Cox proportional hazards regression model. Hazard ratios (HRs) with corresponding 95% confidence intervals (CIs) were calculated to quantify the strength of association. The proportional hazards assumption was evaluated using Schoenfeld residuals, and no significant violation was observed. To address potential multicollinearity among predictors, variance inflation factors (VIFs) were calculated; all VIF values were below 5, indicating acceptable levels of multicollinearity among the included variables. To quantify the potential impact of unmeasured lifestyle and clinical confounders not available in this dataset (e.g., dietary fiber intake, bowel habits, physical activity, family history), *E*-values were calculated for each independent predictor following the method of VanderWeele and Ding (17). The *E*-value represents the minimum strength of association, on the hazard ratio scale, that an unmeasured confounder would need to have with both the omitted factor and hemorrhoid risk, above and beyond the measured covariates, to fully explain away the observed association; *E*-values were calculated for both the point estimate and the confidence interval limit closest to the null. Because current pregnancy could not be directly assessed in this cohort, a sensitivity analysis examined the association between parity and hemorrhoid incidence within the female subgroup. Kaplan–Meier estimates and the log-rank test were used to compare cumulative incidence between parous and nulliparous women, and a multivariable Cox model adjusted for age, HGB, TG, and UA was fitted within this subgroup. The proportional-hazards assumption for parity was evaluated using Schoenfeld residuals; because it was violated, a landmark analysis with a 24-month cut point was additionally performed to estimate hazard ratios separately for the early (0–24 month) and late (24–60 month) follow-up periods.

Based on the independent predictors identified in the multivariate Cox regression analysis, a nomogram was constructed to predict the 60-month probability of new-onset hemorrhoid disease. The nomogram provides a graphical representation of the Cox regression model, enabling clinicians to estimate individualized risk by summing the points assigned to each predictor and mapping the total points to the corresponding predicted probability. Five independent predictors were incorporated into the final nomogram: sex (female), age, hemoglobin (HGB), triglycerides (TG), and uric acid (UA).

All analyses were conducted based on the 60-month follow-up endpoint. This fixed follow-up duration approach, rather than using variable follow-up times, was chosen to standardize risk assessment periods across all participants and enhance interpretability of the nomogram in clinical practice. Participants who did not develop hemorrhoid disease by 60 months were censored at this timepoint, while those who developed hemorrhoids were censored at the time of diagnosis. Participants lost to follow-up before 60 months were censored at their last known contact date.

The predictive performance of the nomogram was evaluated using multiple metrics. Discrimination was assessed by the concordance index (C-index) and the area under the receiver operating characteristic curve (AUC) at 60 months. The C-index quantifies the model's ability to correctly rank pairs of individuals according to their predicted risk, with values ranging from 0.5 (no discrimination) to 1.0 (perfect discrimination).

Calibration was evaluated by comparing the nomogram-predicted probabilities with the observed event rates using calibration curves at 60 months. A well-calibrated model is indicated by close alignment between the predicted and observed probabilities along the 45° reference line. To quantify this graphical assessment, participants with a determinate 60-month outcome status (i.e., those who developed hemorrhoids before 60 months, or who remained event-free with at least 60 months of follow-up; *n* = 14,365) were stratified into deciles of predicted 60-month risk, and the mean predicted probability was compared with the observed event proportion within each decile. The Brier score was calculated as the mean squared difference between predicted probabilities and observed outcomes within this subset. To evaluate whether the linear-term assumption for continuous predictors was appropriate, restricted cubic splines (4 knots, placed at the 5th, 35th, 65th, and 95th percentiles) were fitted for Age, HGB, TG, and UA, and each variable's nonlinear component was tested against the corresponding linear model using a likelihood ratio test. A sensitivity analysis was additionally performed excluding records containing biologically implausible values for BMI, HDL, or creatinine, to confirm that the multivariable model's predictor estimates were not driven by data-entry or measurement outliers.

The clinical utility of the nomogram was assessed using decision curve analysis (DCA), which plots the net benefit across a range of threshold probabilities. DCA enables comparison of the nomogram against the strategies of treating all patients or treating no patients, thereby providing insight into the range of threshold probabilities at which the model offers clinical benefit.

All statistical analyses were performed using R software (version 4.3.1; R Foundation for Statistical Computing, Vienna, Austria). The following R packages were utilized: *survival* for Cox regression analysis, *rms* for nomogram construction and validation, *timeROC* for time-dependent ROC analysis, *rmda* or *ggDCA* for decision curve analysis, and *ggplot2* for data visualization. A two-sided *P* value < 0.05 was considered statistically significant.

## 3 Results

### 3.1 Baseline characteristics of study participants

A total of 35,256 participants were included in the final analysis. During the follow-up period, 3,973 participants (11.27%) developed hemorrhoids. The baseline characteristics of the study population stratified by hemorrhoid incidence status are presented in Table 1.

**Table 1:** Baseline characteristics of the study population.

Variables	Non-incidence (*n* = 31,283)	Incidence (*n* = 3,973)	Statistic	SMD	*P* value
Demographics					
Age (years), Mean ± SD	38.61 ± 11.83	42.80 ± 12.71	*t* = −19.74	0.341	<0.001
Female, *n* (%)	14,377 (45.96)	2,168 (54.57)	*χ*^2^ = 104.94	0.173	<0.001
Smoking, *n* (%)	980 (3.13)	150 (3.78)	*χ*^2^ = 0.46	0.011	0.500
BMI (kg/m^2^), Mean ± SD	24.14 ± 3.40	24.09 ± 3.27	*t* = 0.95	−0.016	0.342
Blood Pressure					
SBP (mmHg), Mean ± SD	122.82 ± 14.71	122.71 ± 15.26	*t* = 0.45	−0.008	0.655
DBP (mmHg), Mean ± SD	75.66 ± 10.15	75.26 ± 10.14	*t* = 2.35	−0.04	0.019
Laboratory Parameters					
HGB (g/L), Mean ± SD	146.25 ± 14.69	144.04 ± 14.65	*t* = 8.91	−0.15	<0.001
WBC (×10^9^/L), Mean ± SD	6.47 ± 1.47	6.38 ± 1.46	*t* = 3.91	−0.066	<0.001
PLT (×10^9^/L), Mean ± SD	254.85 ± 52.84	252.33 ± 54.12	*t* = 2.76	−0.047	0.006
LDL-C (mmol/L), Mean ± SD	2.82 ± 0.62	2.82 ± 0.63	*t* = −0.11	0.002	0.911
Cr (μmol/L), Mean ± SD	65.73 ± 15.28	64.46 ± 13.31	*t* = 5.58	−0.089	<0.001
FPG (mmol/L), Mean ± SD	5.40 ± 1.05	5.36 ± 1.02	*t* = 2.14	−0.036	0.033
TG (mmol/L), Mean ± SD	1.73 ± 1.05	1.70 ± 1.08	*t* = −1.61	−0.027	0.107
ALT (U/L), M (Q_1_, Q_3_)	21.00 (16.00, 28.00)	20.00 (15.00, 27.00)	*Z* = −3.74	−0.049	<0.001
AST (U/L), M (Q_1_, Q_3_)	18.80 (15.80, 24.00)	18.00 (15.00, 23.00)	*Z* = −4.89	−0.076	<0.001
UA (μmol/L), M (Q_1_, Q_3_)	351.80 (296.00, 400.40)	336.00 (279.00, 400.40)	*Z* = −9.37	−0.156	<0.001

BMI, body mass index; DBP, diastolic blood pressure; HGB, hemoglobin; WBC, white blood cell; PLT, platelet; LDL-C, low-density lipoprotein cholesterol; Cr, creatinine; FPG, fasting plasma glucose; TG, triglycerides; ALT, alanine aminotransferase; AST, aspartate aminotransferase; UA, uric acid.

Compared with participants who did not develop hemorrhoids, those who developed hemorrhoids were significantly older (42.80 ± 12.71 vs. 38.61 ± 11.83 years, *P* < 0.001) and more likely to be female (54.57% vs. 45.96%, *P* < 0.001).

Regarding laboratory parameters, participants in the hemorrhoid incidence group had significantly lower hemoglobin levels (144.04 ± 14.65 vs. 146.25 ± 14.69 g/L, *P* < 0.001) and did not differ significantly in triglyceride levels (1.70 ± 1.08 vs. 1.73 ± 1.05 mmol/L, *P* = 0.107); they also showed lower white blood cell counts (6.38 ± 1.46 vs. 6.47 ± 1.47 × 10^9^/L, *P* < 0.001), lower platelet counts (252.33 ± 54.12 vs. 254.85 ± 52.84 × 10^9^/L, *P* = 0.006), lower creatinine levels (64.46 ± 13.31 vs. 65.73 ± 15.28 μmol/L, *P* < 0.001), and slightly lower diastolic blood pressure (75.26 ± 10.14 vs. 75.66 ± 10.15 mmHg, *P* = 0.019).

For non-normally distributed variables, the hemorrhoid incidence group showed lower ALT levels [20.00 (15.00, 27.00) vs. 21.00 (16.00, 28.00) U/L, *P* < 0.001], lower AST levels [18.00 (15.00, 23.00) vs. 18.80 (15.80, 24.00) U/L, *P* < 0.001], and lower uric acid levels [336.00 (279.00, 400.40) vs. 351.80 (296.00, 400.40) μmol/L, *P* < 0.001].

No significant differences were observed between the two groups in terms of smoking status (*P* = 0.500), BMI (*P* = 0.342), TG (*P* = 0.107), or LDL-C (*P* = 0.911).

### 3.2 Risk factors for hemorrhoid incidence

Univariate and multivariable Cox proportional hazards regression analyses were performed to identify independent risk factors for hemorrhoid incidence (Table 2). In the univariate analysis, several variables showed significant associations with hemorrhoid incidence, including sex, age, hemoglobin (HGB), triglycerides (TG), uric acid (UA), white blood cell count (WBC), creatinine (Cr), alanine aminotransferase (ALT), and fasting plasma glucose (FPG) (all *P* < 0.05).

**Table 2:** Association of risk factors with hemorrhoid incidence in Cox proportional hazards regression analysis.

Variables	Univariate					Multivariate				
	*β*	S.E	*Z*	*P*	HR (95% CI)	*β*	S.E	*Z*	*P*	HR (95% CI)
Female										
Male					1.000 (Reference)					1.000 (Reference)
Female	0.356	0.032	11.164	**<0**.**001**	1.428 (1.341–1.520)	0.343	0.047	7.214	**<0**.**001**	1.409 (1.283–1.546)
Smoking										
No					1.000 (Reference)					
Yes	−0.100	0.259	−0.387	0.699	0.905 (0.545–1.502)					
Age	0.022	0.001	18.023	**<0**.**001**	1.023 (1.020–1.025)	0.024	0.001	18.509	**<0**.**001**	1.024 (1.022–1.027)
HGB	−0.011	0.001	−10.762	**<0**.**001**	0.989 (0.987–0.991)	−0.006	0.001	−4.397	**<0**.**001**	0.994 (0.991–0.996)
WBC	−0.034	0.011	−3.093	**0**.**002**	0.967 (0.946–0.988)	−0.017	0.011	−1.503	0.133	0.983 (0.962–1.005)
PLT	0.000	0.000	0.267	0.789	1.000 (1.000–1.001)					
BMI	−0.000	0.005	−0.053	0.958	1.000 (0.991–1.009)					
LDL	0.025	0.024	1.041	0.298	1.025 (0.978–1.075)					
TG	0.039	0.012	3.190	**0**.**001**	1.040 (1.015–1.066)	0.048	0.014	3.464	**<0**.**001**	1.049 (1.021–1.078)
ALT	−0.004	0.001	−4.058	**<0**.**001**	0.996 (0.995–0.998)	−0.002	0.001	−1.783	0.075	0.998 (0.996–1.000)
AST	0.002	0.001	1.448	0.148	1.002 (0.999–1.005)					
Cr	−0.008	0.001	−6.733	**<0**.**001**	0.992 (0.990–0.994)	−0.001	0.001	−0.945	0.345	0.999 (0.996–1.002)
UA	−0.001	0.000	−2.573	**0**.**010**	0.999 (0.999–0.999)	0.001	0.000	4.938	**<0**.**001**	1.001 (1.001–1.002)
FPG	0.041	0.013	3.214	**0**.**001**	1.042 (1.016–1.068)	−0.012	0.015	−0.752	0.452	0.989 (0.959–1.019)
DBP	−0.001	0.002	−0.613	0.540	0.999 (0.996–1.002)					
SBP	−0.001	0.001	−0.633	0.527	0.999 (0.997–1.001)					

BMI, body mass index; DBP, diastolic blood pressure; HGB, hemoglobin; WBC, white blood cell; PLT, platelet; LDL-C, low-density lipoprotein cholesterol; Cr, creatinine; FPG, fasting plasma glucose; TG, triglycerides; ALT, alanine aminotransferase; AST, aspartate aminotransferase; UA, uric acid; HR, hazard ratio, CI, confidence interval.

Bold values indicate statistical significance (*P* < 0.05).

Although diastolic blood pressure (DBP) differed slightly between groups at baseline (75.26 ± 10.14 vs. 75.66 ± 10.15 mmHg, *P* = 0.019; Table 1), it was not significantly associated with hemorrhoid incidence in the univariate Cox regression analysis (HR = 0.999, 95% CI: 0.996–1.002, *P* = 0.540; Table 2) and therefore did not meet the pre-specified entry criterion (*P* < 0.05) for inclusion in the multivariable model. This pattern suggests that the modest baseline difference in DBP reflects its correlation with other baseline characteristics—such as age—that differed between groups, rather than an independent association with hemorrhoid risk.

Variables with *P* < 0.05 in univariate analysis were included in the multivariable Cox regression model. After adjusting for potential confounders, five variables remained independently associated with hemorrhoid incidence: female sex (HR = 1.41, 95% CI: 1.28–1.55, *P* < 0.001), age (HR = 1.02, 95% CI: 1.02–1.03, *P* < 0.001), HGB (HR = 0.99, 95% CI: 0.99–1.00, *P* < 0.001), TG (HR = 1.05, 95% CI: 1.02–1.08, *P* < 0.001), and UA (HR = 1.001, 95% CI: 1.001–1.002, *P* < 0.001). *E*-value sensitivity analysis indicated that the robustness of these associations to unmeasured confounding varied considerably across predictors: female sex (*E*-value = 1.96, based on the confidence interval) and triglycerides (*E*-value = 1.13) were comparatively robust, whereas hemoglobin (*E*-value = 1.07) and uric acid (*E*-value = 1.03) were more sensitive to potential unmeasured confounding. Among female participants (*n* = 16,545), the crude proportions of hemorrhoid incidence were nearly identical between women with and without a history of childbirth (13.07% vs. 13.12%, respectively); however, this comparison is misleading because median follow-up differed substantially between groups (48.9 months for parous women vs. 36.0 months for nulliparous women), giving nulliparous women less opportunity to accumulate follow-up time. Kaplan–Meier estimates, which appropriately account for this differential follow-up, showed consistently and substantially lower cumulative hemorrhoid incidence among parous women at every follow-up time point (12 months: 1.08% vs. 2.46%; 24 months: 3.29% vs. 6.84%; 36 months: 5.82% vs. 10.30%; 48 months: 8.78% vs. 13.43%; 60 months: 11.67% vs. 15.83%; parous vs. nulliparous), and this difference was highly significant by the log-rank test (*χ*^2^ = 33.0, *P* < 0.001) (Supplementary Table S1 and Figure S1). In a multivariable Cox model adjusted for age, HGB, TG, and UA within the female subgroup, parity remained independently associated with lower hemorrhoid risk (HR = 0.82, 95% CI: 0.74–0.90, *P* < 0.001). However, the proportional-hazards assumption was violated for parity (*P* < 0.001), indicating that this protective association was not constant over time. A 24-month landmark analysis confirmed this: the association was strong during the first 24 months of follow-up (HR = 0.50, 95% CI: 0.42–0.61, *P* < 0.001) but essentially absent among participants who remained event-free through 24 months thereafter (HR = 1.01, 95% CI: 0.90–1.14, *P* = 0.82, 24–60 months) (Supplementary Table S1).

Notably, UA showed a directional reversal between the univariate and multivariable analyses: the unadjusted association was modestly inverse (HR = 0.999, 95% CI: 0.999–0.999, *P* = 0.010), whereas the adjusted association was positive (HR = 1.001, 95% CI: 1.001–1.002, *P* < 0.001). This pattern is consistent with confounding or suppression by correlated covariates—such as sex, body mass index, and renal function—that are simultaneously associated with both UA and hemorrhoid incidence; once these covariates are accounted for, UA's independent association emerges as a small but statistically significant positive effect. TG showed a similar pattern: the unadjusted baseline comparison showed no significant difference between groups (*P* = 0.107; Table 1), yet TG remained an independent predictor in the multivariable model (HR = 1.05, *P* < 0.001), consistent with the same type of confounding/suppression effect described above for UA. To assess whether the univariate screening step might have inappropriately excluded other variables with a similar suppression effect, BMI, smoking status, and LDL-C—each excluded due to univariate *P* ≥ 0.05—were individually added to the final multivariable model. None reached statistical significance (BMI: HR = 1.001, *P* = 0.873; smoking: HR = 1.121, *P* = 0.660; LDL-C: HR = 0.953, *P* = 0.055), providing no evidence of a suppression effect analogous to that observed for UA. Standardized mean differences (SMDs) for all baseline variables are reported in Table 1; only age (SMD = 0.341) and female sex (SMD = 0.173) reached conventional thresholds for mild-to-moderate imbalance, while all other variables, including those retained in the final model, showed negligible SMDs (<0.16), indicating that the statistically significant *P* values for several laboratory variables in Table 1 do not reflect materially important between-group differences. BMI showed a similar pattern of apparent null association masking a real underlying relationship: although the univariate linear Cox model showed essentially no association between BMI and hemorrhoid risk (HR = 0.9996, 95% CI: 0.991–1.009, *P* = 0.938), restricted cubic spline analysis revealed a significant inverted-U-shaped relationship (*χ*^2^ = 17.93, df = 2, *P* < 0.001), with risk peaking around a BMI of 24–25 kg/m^2^ and declining at both lower and higher values.

Restricted cubic spline analysis indicated that the linearity assumption was not appropriate for three of the five predictors. Age showed no significant departure from linearity (*χ*^2^ = 4.16, df = 2, *P* = 0.125). In contrast, HGB (*χ*^2^ = 30.28, df = 2, *P* < 0.001), TG (*χ*^2^ = 49.77, df = 2, *P* < 0.001), and UA (*χ*^2^ = 34.41, df = 2, *P* < 0.001) each deviated significantly from a linear relationship with hemorrhoid risk, with non-monotonic patterns detailed in Supplementary Figure S2.

### 3.3 Development of the prediction nomogram

Based on the results of the multivariable Cox regression analysis, a nomogram was constructed to predict the 60-month probability of hemorrhoid incidence (Figure 3). The nomogram incorporated five independent predictors: sex (female), age, HGB, TG, and UA. To use the nomogram, points are assigned for each predictor by drawing a vertical line from each variable value to the top points scale. The total points are calculated by summing the points from all five predictors. The predicted 60-month probability of hemorrhoid incidence can then be determined by drawing a vertical line from the total points axis to the bottom probability scale.

**Figure 3:**
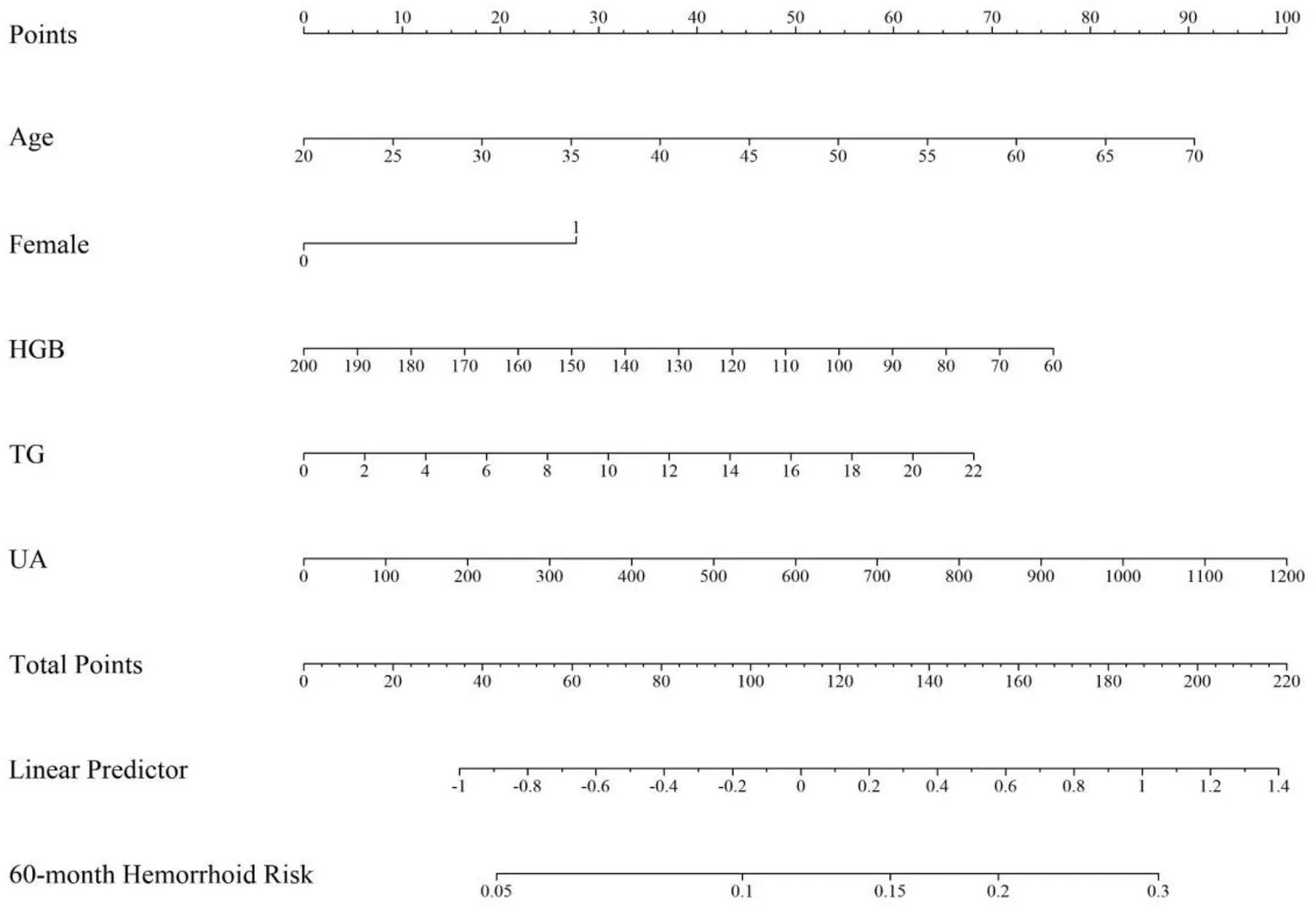
Nomogram for predicting 60-month hemorrhoid incidence risk. Points are assigned for each predictor (sex, age, HGB, TG, and UA), and the total points correspond to the predicted probability of hemorrhoid incidence.

For example, a 55-year-old female patient with HGB of 130 g/L, TG of 1.8 mmol/L, and UA of 350 μmol/L would receive approximately 45 points for female sex, 30 points for age, 15 points for HGB, 8 points for TG, and 12 points for UA, resulting in a total of approximately 110 points. This corresponds to a predicted 60-month hemorrhoid incidence probability of approximately 25%.

### 3.4 Validation of the nomogram

#### 3.4.1 Discrimination

The discriminative ability of the nomogram was evaluated using the concordance index (C-index) and receiver operating characteristic (ROC) curve analysis. The C-index of the nomogram was 0.772 (95% CI: 0.764–0.780), indicating acceptable discriminative ability. The ROC curve analysis further confirmed the discrimination performance of the nomogram (Figure 4). The area under the ROC curve was 0.772 (95% CI: 0.764–0.780), which was consistent with the C-index value and demonstrated the model's ability to discriminate between individuals who developed hemorrhoids and those who did not during the 60-month follow-up period.

**Figure 4:**
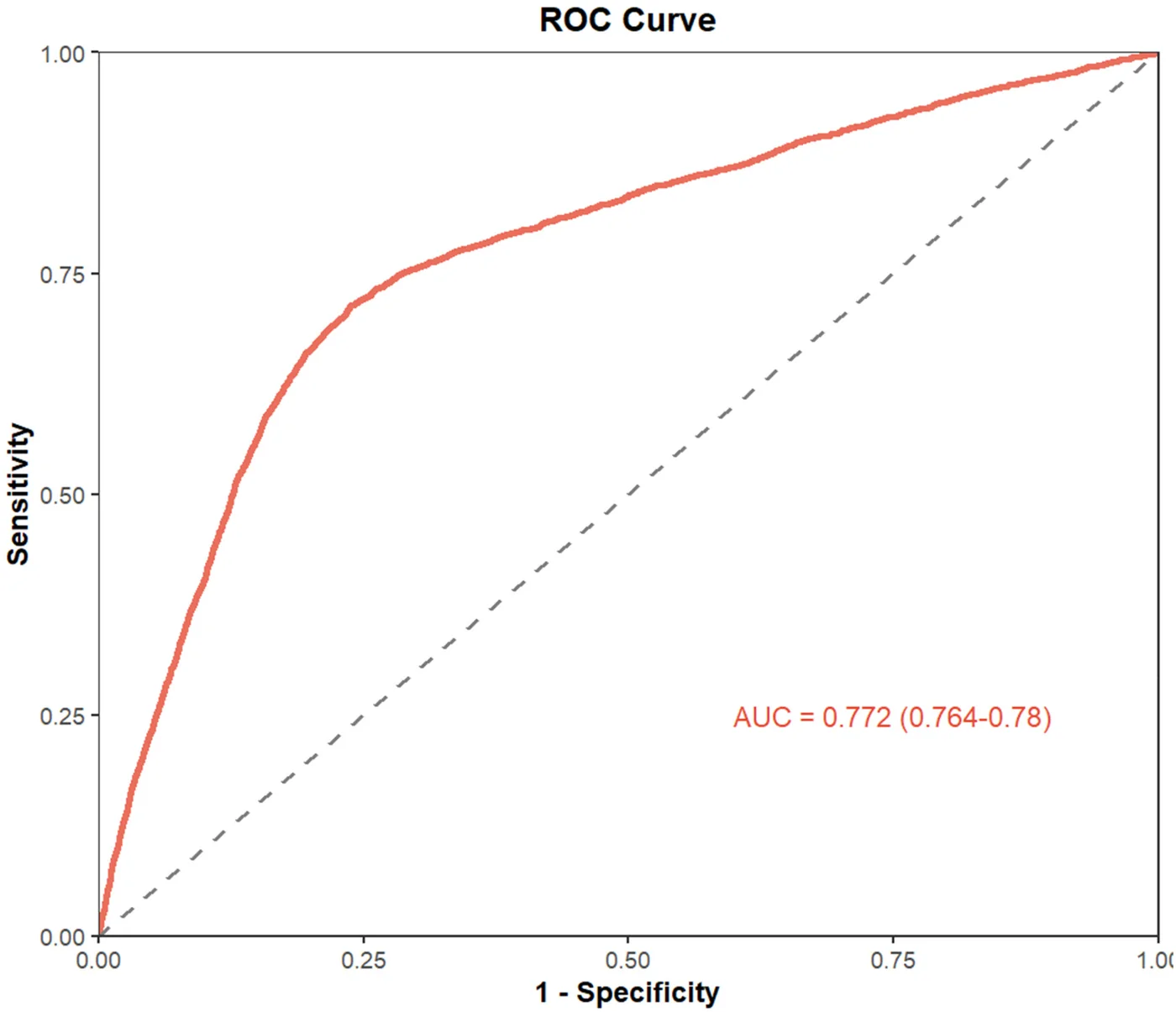
Receiver operating characteristic (ROC) curve of the nomogram prediction model.

#### 3.4.2 Calibration

Calibration curves were generated to assess the agreement between the predicted probabilities and the observed outcomes (Figure 5). Across the range of predicted probabilities covered by the data (approximately 0%–37%), the calibration curve lay consistently above the ideal diagonal line, indicating that the nomogram systematically underestimated the observed 60-month hemorrhoid incidence, with the degree of underestimation increasing at higher predicted-probability levels. This underestimation may partly reflect the linear-term assumption for HGB, TG, and UA, which restricted cubic spline analysis indicated does not hold. This pattern suggests that, while the model retains acceptable discrimination, its absolute risk estimates would benefit from recalibration (e.g., intercept/slope adjustment) before being used to communicate individualized absolute risk to patients or in an external validation cohort. Quantitatively, across the ten deciles of predicted risk, the observed event proportion exceeded the mean predicted probability at every decile, with the gap widening from the lowest to the highest risk stratum (e.g., 10.5% observed vs. 6.9% predicted in the lowest decile, vs. 35.6% observed vs. 22.3% predicted in the highest decile), confirming both the systematic direction and the increasing magnitude of underestimation described above. The overall Brier score was 0.162 (Supplementary Table S2).

**Figure 5:**
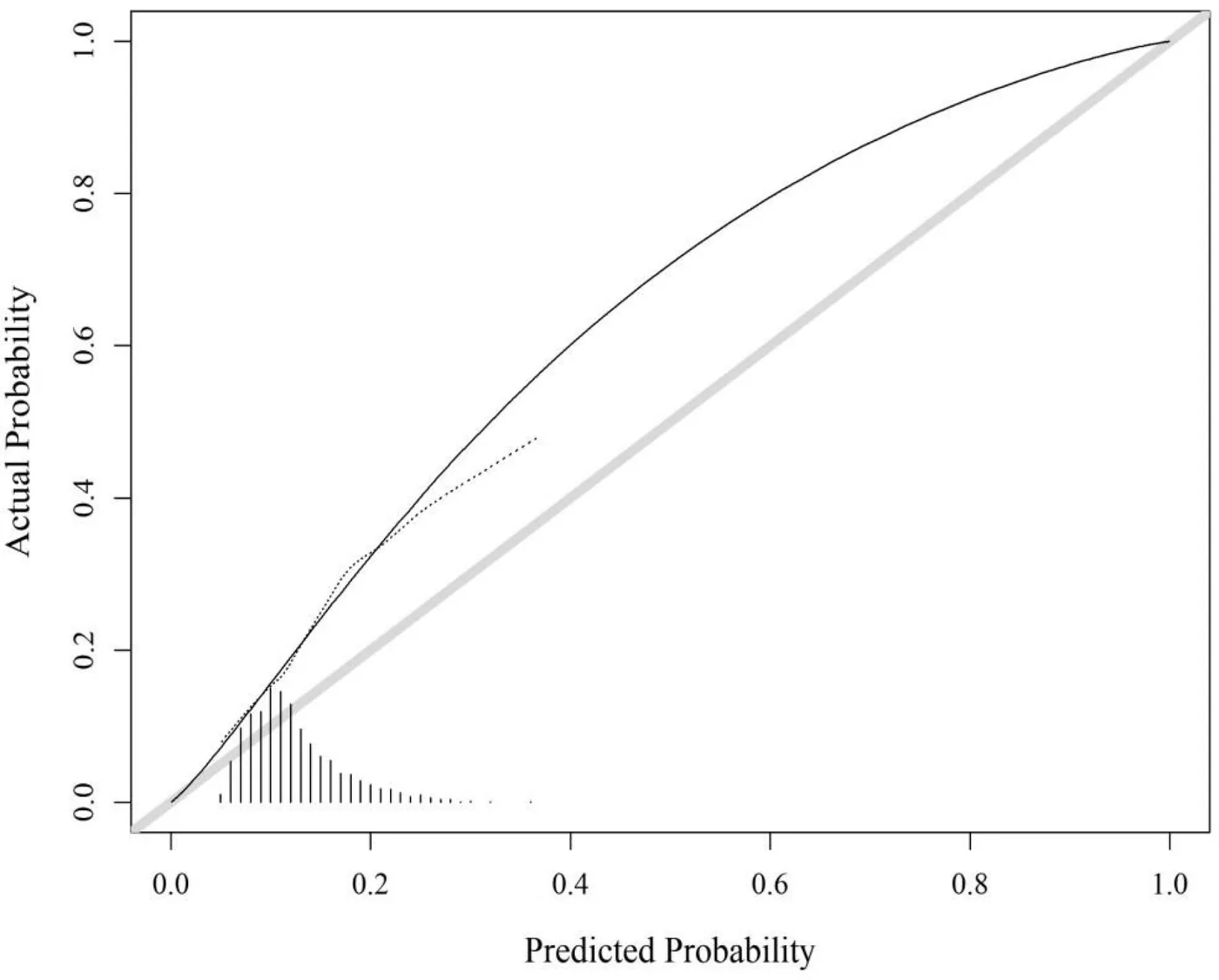
Calibration curve of the nomogram at 60 months. The grey diagonal line represents perfect calibration. The dotted line shows the apparent calibration and the solid black line shows the bias-corrected calibration of the nomogram, which lies above the diagonal across the observed predicted-probability range (approximately 0%-37%), indicating systematic underestimation of observed risk relative to predicted risk; the rug plot along the x-axis shows the distribution of predicted probabilities in the validation sample.

#### 3.4.3 Clinical utility

Decision curve analysis (DCA) was performed to evaluate the clinical utility of the nomogram (Figure 6). The overall event rate was 11.3%. The DCA demonstrated that using the nomogram to predict hemorrhoid incidence would provide greater net benefit compared with both the “treat all” and “treat none” strategies across a range of threshold probabilities from approximately 5% to 40%, encompassing the risk levels at which clinicians and patients might reasonably consider initiating preventive interventions or lifestyle modifications. Importantly, the model's net benefit curve remained consistently at or above the “treat none” line across the entire threshold range examined, suggesting that the use of the nomogram would not result in net harm compared to a strategy of no intervention.

**Figure 6:**
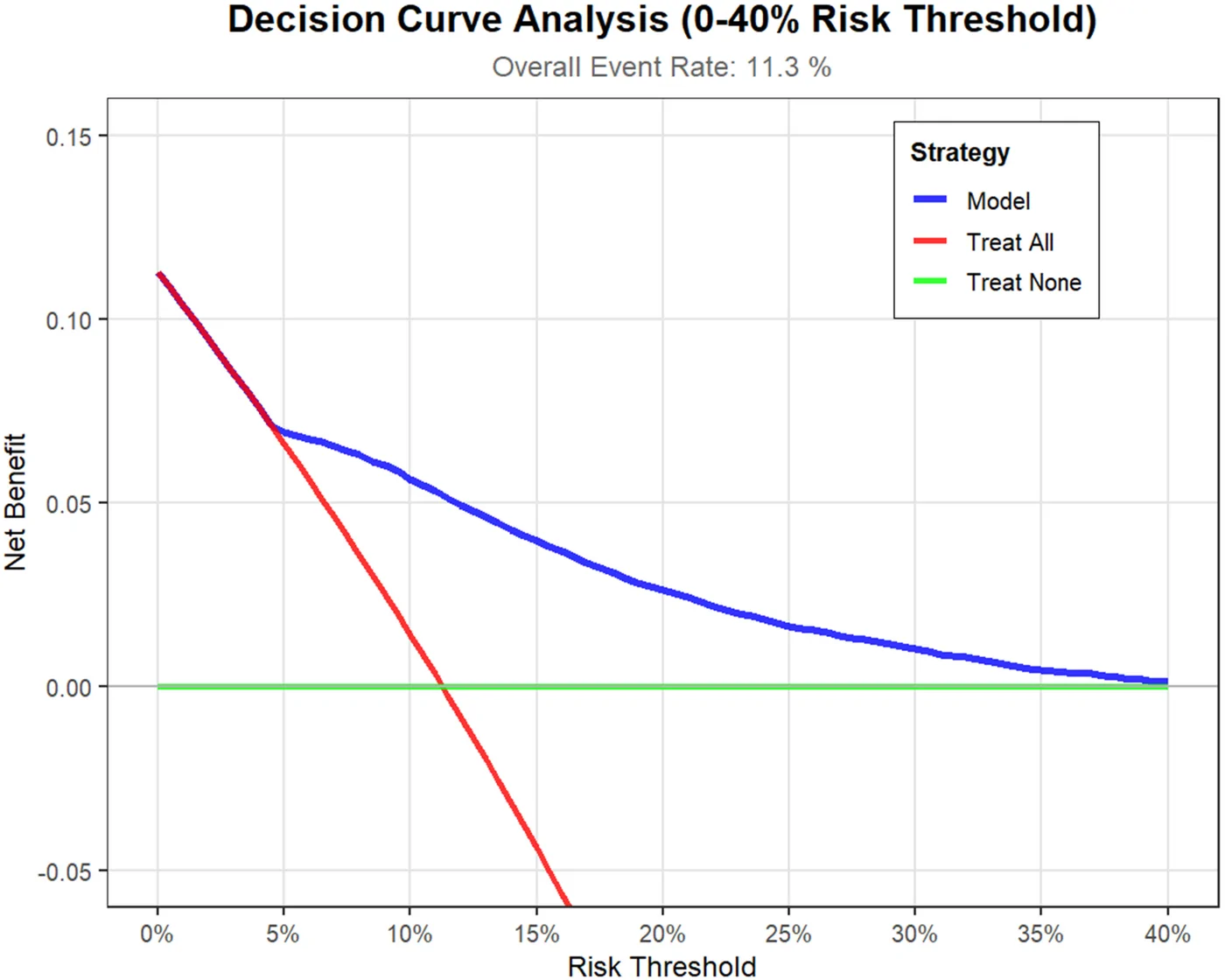
Decision curve analysis (DCA) of the nomogram at 60 months. The red line represents the assumption that all patients develop hemorrhoids (“treat all”), and the horizontal green line represents the assumption that no patients develop hemorrhoids (“treat none”).

## 4 Discussion

This study developed and internally validated a predictive nomogram for hemorrhoid incidence using routine health-screening parameters in a large cohort of 35,256 adult participants followed for 60 months. Five independent predictors were identified: female sex (HR = 1.41, 95% CI: 1.28–1.55, *P* < 0.001), older age (HR = 1.02 per year, 95% CI: 1.02–1.03, *P* < 0.001), lower hemoglobin (HR = 0.99, 95% CI: 0.99–1.00, *P* < 0.001), higher triglycerides (HR = 1.05, 95% CI: 1.02–1.08, *P* < 0.001), and higher uric acid (HR = 1.001, 95% CI: 1.001–1.002, *P* < 0.001). The nomogram demonstrated acceptable discriminative ability with a C-index of 0.772 (95% CI: 0.764–0.780), though calibration analysis indicated that the model systematically underestimated observed hemorrhoid incidence relative to predicted risk across the range covered by the data (approximately 0%–37%). Decision curve analysis revealed positive net benefit for threshold probabilities between approximately 5%–40%, demonstrating clinical utility for individualized risk stratification. To our knowledge, this is among the first studies to develop a nomogram integrating routine laboratory biomarkers for hemorrhoid risk prediction in a large health-screening cohort, enabling accessible, low-cost risk assessment using parameters already available in standard screening settings.

Female sex predisposition and age have been documented as hemorrhoid risk factors in epidemiological studies and genome-wide association analyses. A recent systematic review and meta-analysis reported higher hemorrhoid prevalence in women (27.33%, 95% CI: 21.84–32.82) compared to men, with older age identified as a significant risk factor in unadjusted analyses (1). Cross-sectional studies in Korean adults demonstrated higher hemorrhoid prevalence in females than males (17.2% vs. 16.3%, *P* < 0.001), with older age independently associated with hemorrhoid presence (5). The current study quantifies these associations longitudinally, demonstrating that females had 41% higher risk of hemorrhoid development (HR = 1.41, 95% CI: 1.28–1.55, *P* < 0.001) and each 1-year age increase conferred 2% increased risk (HR = 1.02, 95% CI: 1.02–1.03, *P* < 0.001). While prior studies identified sex and age as categorical risk factors, our study provides precise hazard ratios and continuous risk quantification in a large health-screening cohort with longitudinal follow-up. The female predisposition reflects biological mechanisms including pregnancy-related pelvic floor changes, hormonal influences on vascular tone, and anatomical differences in anal sphincter morphology and anorectal motion (18). Because this occupational cohort structurally excludes pregnant women, the female-sex effect observed here necessarily reflects non-pregnancy-related mechanisms rather than the acute effects of current pregnancy; pregnancy itself is a well-established independent risk factor for hemorrhoidal disease through increased pelvic venous pressure and progesterone-mediated venous dilation (19). We were, however, able to examine parity as an available proxy for reproductive history, and found it was associated with a substantially lower hemorrhoid risk that was concentrated in the first 24 months of follow-up and essentially absent thereafter. This time-limited pattern is unlikely to reflect a stable, lifelong protective effect of childbirth; parity in this dataset captures only whether a woman had ever given birth, not the time elapsed since her most recent delivery. We hypothesize that hemorrhoidal disease arising in the peripartum period is common but often resolves within the following months (20), and that women who had given birth relatively recently at cohort entry may therefore have had their postpartum hemorrhoidal symptoms identified and treated within that window, reducing the likelihood of a new diagnosis being captured at the next annual screening visit; as time since delivery lengthens across the parous group, this transient effect would be expected to fade, consistent with the observed pattern. This explanation remains a hypothesis rather than a confirmed mechanism, as exact delivery-date data were not available in this dataset. Age-related risk accumulates through cumulative vascular degeneration, progressive weakening of anal cushion supportive connective tissue, and chronic straining over the lifespan. Previous literature focused on prevalence estimates and categorical associations rather than continuous risk quantification, which is critical because individualized risk stratification requires precise, continuous risk increments applicable across the age spectrum. The current study addresses this by providing quantifiable, continuous hazard ratios suitable for integration into a nomogram, enabling personalized risk assessment at routine health screening.

Laboratory biomarkers including hemoglobin, triglycerides, and uric acid have received limited attention as hemorrhoid predictors in prior literature, representing a potentially novel contribution of this study. The current study identified three independent laboratory predictors: lower hemoglobin (HR = 0.99, 95% CI: 0.99–1.00, *P* < 0.001), higher triglycerides (HR = 1.05, 95% CI: 1.02–1.08, *P* < 0.001), and higher uric acid (HR = 1.001, 95% CI: 1.001–1.002, *P* < 0.001). The inverse association between hemoglobin and hemorrhoid risk presents a complex interpretive challenge due to potential reverse causation (21). Lower hemoglobin may reflect chronic subclinical bleeding from pre-existing but undiagnosed hemorrhoidal vessels, representing a disease marker rather than a true etiological factor. Alternatively, hemoglobin deficiency could genuinely precede hemorrhoid development through mucosal hypoxia impairing tissue repair capacity and vascular integrity. The study design does not definitively resolve this directionality issue, as baseline hemoglobin levels may already reflect occult hemorrhoidal pathology (22). From a clinical standpoint, this distinction matters for how the finding should be used: if reduced hemoglobin chiefly reflects pre-existing, undiagnosed hemorrhoidal bleeding rather than a true risk factor, a low hemoglobin value identified at screening should prompt consideration of existing hemorrhoidal disease and further clinical evaluation—not be interpreted as a modifiable target whose correction would itself lower hemorrhoid risk. Elevated triglycerides link to metabolic syndrome, endothelial dysfunction, and chronic low-grade inflammation (23), metabolic disturbances that promote anal cushion vascular degeneration and impaired tissue repair (24). Dyslipidemia-associated oxidative stress and inflammatory cytokines may compromise vascular integrity in the hemorrhoidal plexus. Uric acid exhibits dual roles as antioxidant and pro-oxidant; elevated uric acid promotes oxidative stress, reduces nitric oxide bioavailability, activates the renin-angiotensin system, and induces vascular smooth muscle proliferation (12). These mechanisms connect to hemorrhoidal vascular pathology through vessel wall thickening, reduced vascular compliance, and impaired venous return. Previous literature focused on demographic and lifestyle risk factors rather than routine laboratory biomarkers, which is critical because laboratory parameters are objectively measured, widely available in health screening, and reflect underlying metabolic and hematological pathophysiology. The current study addresses this gap by systematically integrating routine laboratory biomarkers into hemorrhoid risk prediction, bridging metabolic and hematological parameters with hemorrhoid pathophysiology and providing a novel, accessible approach to risk stratification. The apparent absence of a BMI association in this cohort, which contrasts with prior literature identifying obesity as a hemorrhoid risk factor, is likewise attributable to its non-linear, inverted-U relationship with risk rather than a true absence of association; a linear model necessarily averages the opposing effects on either side of the peak, yielding a near-null estimate. The rising portion of this curve, from underweight to a BMI of approximately 24–25 kg/m^2^, is consistent with early metabolic and vascular changes that accompany increasing adiposity, whereas the declining risk at higher BMI values may reflect a healthy-worker effect in this occupational screening cohort, in which individuals with more severe obesity and associated comorbidities are underrepresented among adults who remain actively employed and undergo routine health screening. Screening for physiologically implausible values identified 19 records with BMI <15 kg/m^2^ (*n* = 8), HDL <0.3 mmol/L (*n* = 9), or creatinine >500 μmol/L (*n* = 2). Excluding these records and refitting the multivariable model produced materially unchanged hazard ratios for all five predictors (Supplementary Table S3), confirming that the primary results were not driven by extreme or implausible values.

The nomogram demonstrated a C-index of 0.772 (95% CI: 0.764–0.780), indicating acceptable discrimination for a screening tool using only routine parameters. This performance is appropriate for population-level risk stratification using accessible, low-cost biomarkers without additional testing burden. A model with this level of discrimination may still provide clinical utility when the predicted risks fall within the range where clinical decisions are uncertain, providing sufficient information to guide risk stratification decisions (25). The model is designed to identify individuals at elevated risk who may benefit from targeted preventive counseling, not to replace clinical examination or confirm hemorrhoid diagnosis. Calibration is critically important for screening models where individualized risk estimates inform preventive interventions (26). However, calibration analysis in the present study revealed that the nomogram systematically underestimated observed hemorrhoid incidence relative to predicted risk, particularly at higher predicted-probability levels. Decision curve analysis demonstrated positive net benefit for threshold probabilities between approximately 5%–40%, confirming clinical utility across clinically relevant risk thresholds for screening applications. Similar nomogram models using routine parameters for metabolic syndrome prediction in screening populations have employed comparable methodological validation approaches combining AUC metrics with decision curve analysis to establish clinical utility (27), with discrimination in a comparable range to the present model, supporting the feasibility of routine-biomarker-based nomograms for population-level screening applications. The practical advantages of this model include exclusive use of routine, low-cost parameters available in standard health screening, eliminating additional testing burden or cost. The nomogram enables individualized risk stratification at the point of routine health screening, facilitating early lifestyle intervention for high-risk individuals. Clinical applications include targeted surveillance and preventive counseling regarding dietary fiber intake, hydration, and bowel habits for individuals with elevated predicted risk. As a screening tool appropriate for population-level risk stratification rather than definitive diagnosis, the nomogram complements clinical assessment and positions hemorrhoid risk prediction within the framework of routine preventive health screening.

This study has several limitations that warrant consideration. The single-center retrospective design limits generalizability, with potential selection bias from a health-screening population that may not represent the general population. The lack of external validation cohort necessitates confirmation of model performance and calibration in independent populations from different geographic and demographic settings, as recommended by methodological guidance for clinical prediction model evaluation (28). Some established hemorrhoid risk factors were not available in the dataset, including dietary fiber intake, bowel habits, physical activity, constipation history, and family history, factors identified as associated with hemorrhoid development in prior studies (1, 5). The female-sex and triglyceride associations proved comparatively robust to this omission, exceeding the strength of confounding plausible for dietary fiber intake, physical activity, and sedentary behavior reported in large screening-based cohorts (odds ratios approximately 0.78–0.83) (6). The hemoglobin and uric acid associations, however, were considerably more fragile, meaning that even a weak unmeasured lifestyle or clinical confounder could plausibly account for these findings in whole or in part; these two predictors should therefore be interpreted with appropriate caution until validated in cohorts with more complete lifestyle and clinical data. The absence of these lifestyle and clinical variables may partially explain the acceptable C-index; incorporating these factors could improve predictive accuracy. In addition, parity captures only whether a woman had ever given birth, not the timing of her most recent delivery or whether she was pregnant during the study period itself; consequently, the acute effects of current pregnancy on hemorrhoid risk remain unassessed in this cohort. Hemorrhoid diagnosis relied on structured surgical department examination findings, supplemented by outpatient and inpatient medical-record linkage; this approach has imperfect sensitivity for internal hemorrhoids without external prolapse, and anoscopy was not routinely performed, which may have led to underdiagnosis of mild or early-stage asymptomatic disease. In addition, because examination documentation records abnormal findings selectively, it was not possible to confirm from the available records that a perianal examination was performed and explicitly documented at every screening visit for every participant. The nomogram also assumes linear relationships between continuous predictors and risk; this assumption did not hold for HGB, TG, and UA. We evaluated a nonlinear alternative but retained the linear specification for the primary nomogram due to reduced clinical interpretability of the resulting point-scoring axes. Future research should prioritize multi-center prospective validation studies to confirm model generalizability across diverse populations. Incorporating lifestyle variables including dietary fiber, physical activity, and bowel habits, along with clinical factors such as constipation and family history, may enhance model performance. Integration with genetic markers from genome-wide association studies could enable development of comprehensive risk prediction models combining genetic, metabolic, and lifestyle factors (4). Randomized controlled trials investigating whether nomogram-guided interventions improve hemorrhoid prevention outcomes would establish clinical effectiveness and inform implementation strategies.

## 5 Conclusions

This study developed one of the first nomograms to integrate routine laboratory biomarkers—hemoglobin, triglycerides, and uric acid—alongside sex and age for hemorrhoid risk prediction. Using exclusively accessible, low-cost parameters, the model enables individualized risk stratification at routine health screening, supporting targeted preventive counseling for high-risk individuals in primary care settings.

## Data Availability

The datasets presented in this article are not readily available because of patient privacy considerations. Requests to access the datasets should be directed to the corresponding author, Li Yan, 3911100886@163.com.
