# Supplementary material for: A clinical prediction nomogram for hemorrhoid risk in health screening populations in Beijing, China: a retrospective cohort study of 35,256 adults
Source: Front Med (Lausanne). 2026 Sep 28;13:1846099. doi: 10.3389/fmed.2026.1846099 (PMC13655133; doi:10.3389/fmed.2026.1846099)
Supplement: Supplementary file 1 [file Datasheet1.docx]

**Supplementary Materials**

**Supplementary Table S1. Sensitivity analysis of parity and hemorrhoid risk among female participants (n = 16,545)**

(a) Kaplan-Meier cumulative incidence of hemorrhoid disease by parity status

| **Follow-up (months)** | **Nulliparous, n at risk** | **Nulliparous, cumulative incidence (95% CI)** | **Parous, n at risk** | **Parous, cumulative incidence (95% CI)** |
| --- | --- | --- | --- | --- |
| 12 | 10,851 | 2.5% (2.2–2.7%) | 4,018 | 1.1% (0.8–1.4%) |
| 24 | 8,138 | 6.8% (6.4–7.3%) | 3,449 | 3.3% (2.7–3.8%) |
| 36 | 6,132 | 10.3% (9.7–10.9%) | 2,851 | 5.8% (5.0–6.6%) |
| 48 | 4,602 | 13.4% (12.7–14.2%) | 2,226 | 8.8% (7.8–9.8%) |
| 60 | 3,497 | 15.8% (15.0–16.7%) | 1,812 | 11.7% (10.5–12.8%) |

*Log-rank test: χ² = 33.0, df = 1, P < 0.001.*

(b) Multivariable Cox regression within female participants

| **Variable** | **HR** | **95% CI** | **P** |
| --- | --- | --- | --- |
| Age | 1.027 | 1.024–1.030 | <0.001 |
| HGB | 0.997 | 0.993–1.000 | 0.078 |
| TG | 1.065 | 1.015–1.119 | 0.011 |
| UA | 1.0003 | 0.9996–1.0010 | 0.366 |
| Parity | 0.820 | 0.744–0.904 | <0.001 |

*Proportional-hazards test (Schoenfeld residuals): Parity χ² = 53.7, P < 0.001 (assumption violated).*

(c) 24-month landmark analysis, Parity (adjusted for Age/HGB/TG/UA)

| **Follow-up segment** | **N** | **HR** | **95% CI** | **P** |
| --- | --- | --- | --- | --- |
| 0–24 months | 16,545 | 0.503 | 0.416–0.607 | <0.001 |
| 24–60 months (landmark survivors) | 11,587 | 1.014 | 0.901–1.140 | 0.821 |

**Supplementary Table S2. Quantitative calibration of the nomogram at 60 months, by decile of predicted risk**

| **Decile of predicted risk** | **N** | **Mean predicted probability** | **Observed event proportion** |
| --- | --- | --- | --- |
| 1 (lowest) | 1,437 | 0.069 | 0.105 |
| 2 | 1,436 | 0.082 | 0.142 |
| 3 | 1,437 | 0.094 | 0.143 |
| 4 | 1,436 | 0.104 | 0.150 |
| 5 | 1,437 | 0.113 | 0.166 |
| 6 | 1,438 | 0.122 | 0.183 |
| 7 | 1,434 | 0.133 | 0.218 |
| 8 | 1,437 | 0.148 | 0.254 |
| 9 | 1,436 | 0.172 | 0.295 |
| 10 (highest) | 1,437 | 0.223 | 0.356 |

**Supplementary Table S3. Sensitivity analysis excluding records with physiologically implausible values (n = 19)**

| **Predictor** | **HR (full cohort, n=35,254)** | **HR (extremes excluded, n=35,235)** |
| --- | --- | --- |
| Age | 1.024 | 1.024 |
| Female | 1.437 | 1.435 |
| HGB | 0.993 | 0.993 |
| TG | 1.041 | 1.045 |
| UA | 1.0011 | 1.0011 |

**Supplementary Figure S1. Kaplan-Meier cumulative incidence curves of hemorrhoid disease by parity status among female participants, with number-at-risk table (log-rank P<0.0001).**

Supplementary Figure S2. Restricted cubic spline curves for hemoglobin, triglycerides, and uric acid (hazard ratio vs. value, reference = median; 4-knot spline).

**Supplementary Figure S3. Nonlinear nomogram (hemoglobin, triglycerides, and uric acid as restricted cubic splines), illustrating the axis readability issue underlying retention of the linear nomogram (Figure 3).**
